# Application of stem cells in the repair of intervertebral disc degeneration

**DOI:** 10.1186/s13287-022-02745-y

**Published:** 2022-02-11

**Authors:** Wentao Zhang, Tianze Sun, Ying Li, Ming Yang, Yantao Zhao, Jing Liu, Zhonghai Li

**Affiliations:** 1 Department of Orthopedics, First Affiliated Hospital of Dalian Medical University, Dalian, People’s Republic of China; 2 Key Laboratory of Molecular Mechanism for Repair and Remodeling of Orthopedic Diseases, Dalian, People’s Republic of China; 3 Department of Orthopedics, Fourth Medical Center of PLA General Hospital, Beijing, People’s Republic of China; 4 Stem Cell Clinical Research Center, National Joint Engineering Laboratory, First Affiliated Hospital of Dalian Medical University, Dalian, People’s Republic of China; 5 Dalian Innovation Institute of Stem Cell and Precision Medicine, Dalian, People’s Republic of China

**Keywords:** Intervertebral disc, Intervertebral disc degeneration, Stem cell, Therapy, Repair, Reverse degeneration

## Abstract

Intervertebral disc degeneration (IDD) is a common disease that increases with age, and its occurrence is stressful both psychologically and financially. Stem cell therapy for IDD is emerging. For this therapy, stem cells from different sources have been proven in vitro, in vivo, and in clinical trials to relieve pain and symptoms, reverse the degeneration cascade, delay the aging process, maintain the spine shape, and retain mechanical function. However, further research is needed to explain how stem cells play these roles and what effects they produce in IDD treatment. This review aims to summarize and objectively analyse the current evidence on stem cell therapy for IDD.

## Introduction

With the acceleration of population aging, the incidence of spinal degenerative diseases has increased significantly, and the main sign is chronic low back pain, which seriously affects patients’ quality of life and increases the economic burden on their family and society. Although the aetiologies of spinal degenerative diseases are varied and complex, intervertebral disc degeneration (IDD) is recognized as one of the most important causes. Degenerative disc diseases (DDDs) arising from IDD comprise a series of painful spinal diseases that include discogenic low back pain and lumbar disc herniation [1]. At present, most patients use rest or conservative treatment for pain relief, as well as a variety of drugs such as steroids, local anaesthetics, and other blocking agents. When these methods are ineffective, surgery is often performed to relieve symptoms and improve quality of life. Surgical treatments can also solve pain problems, but have disadvantages such as inability to replace decreased nucleus pulposus (NP) cells, inability to reverse the pathological state of the intervertebral disc (IVD), and potential to cause various intraoperative and postoperative complications [2–4].

In recent years, with the rapid development of stem cell technologies that have been effectively applied in haematology, circulation, orthopaedics, and other fields [5–7], stem cells have attracted the attention of researchers and clinicians. With in-depth studies on the IVD and IDD as well as its mechanism, many teams have found that combination of stem cell technology and treatment for IDD can not only maintain the normal physiological function and structure of the IVD, but even reverse the IDD cascade [8, 9]. Organic combination of the IVD and stem cell technology has outstanding advantages for IDD treatment and recovery, but remains controversial [10–12]. The present review aims to provide an overview of the current advances in stem cell therapy for IDD and to discuss the limitations of different cell applications and the future challenges of this technology.

## IVD and IDD

A normal IVD consists of the NP, annulus fibrosus (AF), and upper and lower cartilaginous endplates (CEPs) [13]. The NP is tightly enclosed by the circular AF that consists of water, NP cells, notochord cells (NCs), and extracellular matrix (ECM). The NP is highly hydrated and its main function is to resist pressure from the spine. The AF is a concentric layered structure with 15–25 layers mainly composed of collagen (COL) I and an outer layer composed of fibroblasts with neurovascular distribution [14]. The CEPs are horizontal hyaline cartilaginous structures that act as important structures for the IVD to carry out substance exchange and information transfer. The ECM is mainly composed of COL II, fibres, elastin, and large amounts of proteoglycans, especially aggrecan (ACAN), with negative charge [15].

**Fig. 1:**
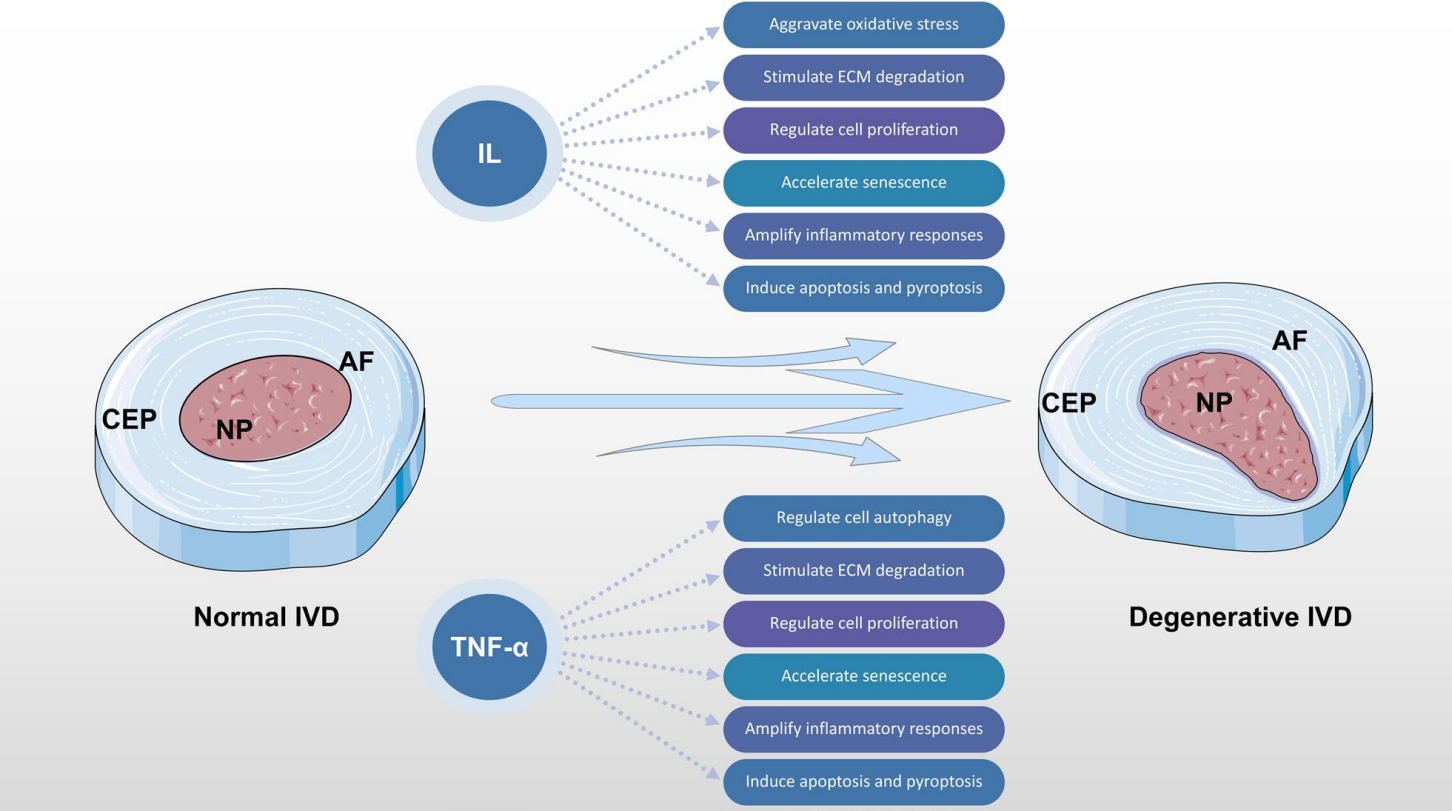
IL and TNF-α are involved in multiple pathological processes of IDD. *AF* annulus fibrosus, *CEP* cartilaginous endplates, *ECM* extracellular matrix, *IL* interleukin, *IVD* intervertebral disc, *NP* nucleus pulposus, *TNF* tumor necrosis factor

When IDD occurs, the NP can break through the posterior edge of the AF and contact the epidural space under the action of external forces. The antigenicity of the NP itself coupled with the inflammatory reaction caused by structural changes can stimulate the immune system to produce pain and discomfort that affect both the body and the mind. Studies have shown that prominent IVD tissue has a variety of inflammatory cell responses with important links to the IDD process. Interleukins (ILs) can regulate the activity of matrix metalloproteinases (MMPs), inhibit the synthesis of proteoglycans in the cell matrix, participate in the IDD process, and contribute to the processes of disc herniation and degeneration by regulating the production of matrix-degrading enzymes by immune cells. Tumour necrosis factor (TNF)-α, a strong inflammatory factor, can up-regulate expression of MMP genes, stimulate production of IL-6, IL-8, and other related cytokines, promote cell migration, affect endothelial cell permeability to block collagen and proteoglycan synthesis, and induce inflammatory reactions [16–18] (Fig. 1).

Recent studies have suggested that NP cell reduction and ECM degradation form the important basis of IDD, and that its occurrence is related to massive release of pro-inflammatory cytokines. In early IDD, the number of NP cells decreases, the activity of NP cells is inhibited, and the secretion of proteoglycans decreases. As the disease progresses, the IVD water content decreases, the NP tissue fibres harden, and the spinal biomechanics change, resulting in arthritis of surrounding tissues and formation of bone spurs. In the later stages of IDD, symptoms of nerve compression can occur, and severe cases may develop spinal cord stenosis, spondylolisthesis, and degenerative scoliosis [19, 20].

Because the IVD has no blood vessels, its internal and external material exchanges can only occur through concentration gradients of these materials. Therefore, it is difficult to achieve healing of IDD through self-repair, and IDD exhibits the characteristics of irreversible injury [13].

## Types and functions of stem cells

Stem cells are abundant and easy to obtain, have very low immunogenicity and strong ability to induce differentiation, and can proliferate in low oxygen and low glucose environments. At present, stem cells have been applied in the treatment of many diseases in various fields [21–24].

Stem cells have been used to intervene in the IDD process to slow down IDD progression or achieve IVD repair. Stem cells currently used in IVD treatment include mesenchymal stem cells (MSCs), intervertebral disc-derived stem cells (IVDSCs), and pluripotent stem cells (PSCs). MSCs include bone marrow mesenchymal stem cells (BMSCs), adipose-derived mesenchymal stem cells (ADMSCs), and umbilical cord mesenchymal stem cells (UCMSCs), IVDSCs include nucleus pulposus stem cells (NPSCs), annulus fibrosus stem cells (AFSCs), and cartilage endplate stem cells (CESCs), and PSCs include induced pluripotent stem cells (IPSCs) and embryonic stem cells (ESCs) (Table 1).

**Table 1:** Sources of stem cells for disk regeneration

Cell types	Source	Advantages	Disadvantages
*MSCs*			
BMSCs	*Bone marrow*	Strong self-renewal ability, multiple differentiation potential, with homing ability, and technology for solation and expansion is mature	The way obtaining BMSCs is invasive
ADMSCs	Adipose	Abundance, ease to harvest, low immunogenicity	Poor ability to differentiate into chondrocytes
UCMSCs (WJMSCs)	Umbilical cord	Pluripotent, with no ethical barriers, strong proliferation ability, extensive differentiation ability, low immunogenicity and no tumorigenicity	Almost impossible to obtain autologous cord cells, and the experimental cost of WJMSCs is high
IVDSCs	IVD	Can be stimulated to proliferate and differentiate in situ	Low yield in number, decreased viability, and expression of proteoglycan and COL II in IDD, and the curative effect is not obvious
*PSCs*			
IPSCs	Artificially derived from somatic cells by reprogramming with transcription factors	High capacities of self-renewal, proliferation, and differentiation	Safety problems, especially potential tumorigenicity
ESCs	Early-stage embryo	High capacities of self-renewal, proliferation, and differentiation	Ethical barriers

*ADMSCs* adipose mesenchymal stem cells, *BMSCs* bone marrow mesenchymal stem cells, *COL II* collagen type II, *ESCs* embryonic stem cells, *IDD* intervertebral disc degeneration, *IPSCs* induced pluripotent stem cells, *IVD* intervertebral disc, *IVDSCs* intervertebral-derived stem cells, *MSCs* mesenchymal stem cells, *PSCs* pluripotent stem cells, *UCMSCs* umbilical cord mesenchymal stem cells, *WJMSCs* Wharton's Jelly mesenchymal stem cells

**Fig. 2:**
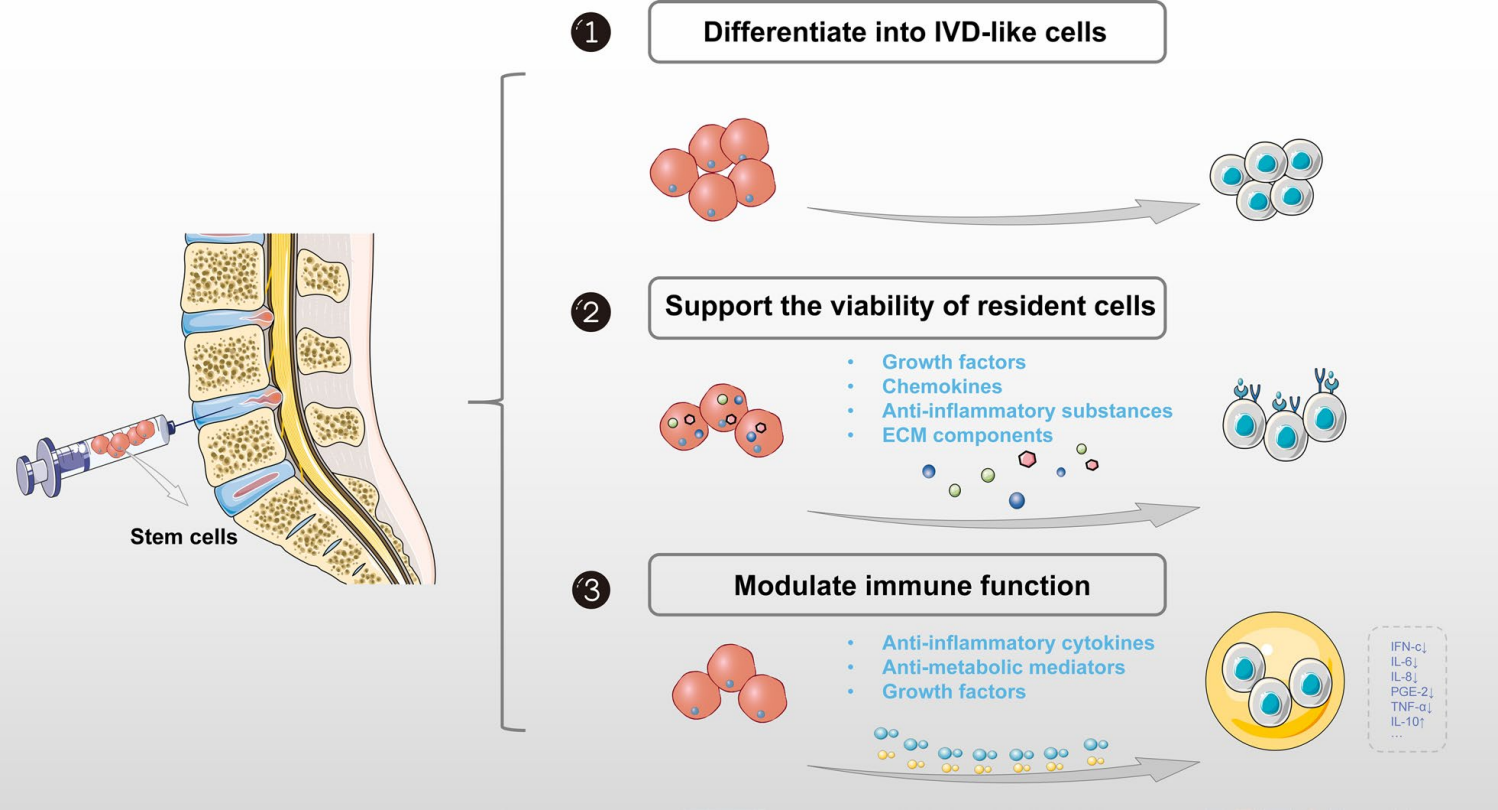
The main functions for stem cells in the context of degenerative IVD. *ECM* extracellular matrix, *IFN* interferon, *IL* interleukin, *IVD* intervertebral disc; *PGE* prostaglandin E, *TNF* tumor necrosis factor

There are three main functions for stem cells in the context of the degenerative IVD. First, stem cells can differentiate into IVD-like cells. The most promising regenerative effects for IVD implantation of stem cells are their differentiation ability into NP cells and their synthesis of new ECM. Although the survival time of cells in the harsh IVD environment or even worse IDD environment remains controversial [25–27], some reports indicated that stem cells can die soon after transplantation because of the effects of nutrition deficiency and pH, while other reports described that MSCs and their progenitors can survive in the long term after injection as either large cell clusters and solitary cells. Second, stem cells can support the viability of resident cells at the implantation site. Many preclinical studies showed that stem cells can stimulate resident IVD cells by secreting certain growth factors, chemokines, ECM components, and anti-inflammatory substances through paracrine mechanisms. In addition to the increased ECM secretion, the expression of proteases related to cell senescence showed downward trends in co-cultures of stem cells and NP cells [28, 29]. At the same time, stem cells can directly change the mechanical properties of NP cells and reduce the hardness of the cells and matrix to support the survival of NP cells [30–32]. Third, the IDD process can be slowed down by immune regulation. During IDD, ECM destruction is accelerated by increased pro-inflammatory cytokines that promote chemotaxis, angiopoietins, and additional cytokine release to maintain an ongoing inflammatory response mediated by ILs, TNFs, interferon (IFN)-c, prostaglandin E2 (PGE-2), and other chemokines. These processes act together on cells, leading to further apoptosis, senescence, and autophagy. Studies have shown that when stem cells are co-cultured in an IDD-like environment in vivo or in vitro, they can produce anti-inflammatory cytokines, anti-metabolic mediators, and growth factors, thus modulating the immune response [33–35] (Fig. 2).

## In vitro, in vivo, and clinical applications of stem cells in IDD

### BMSCs

BMSCs are non-hematopoietic stem cells in the bone marrow that possess self-renewal ability, multiple differentiation potential, and certain homing ability, and can enhance the survival and regeneration of IVD cells and prevent further deterioration of IDD [36]. BMSCs can repair degenerated IVD tissues by promoting cell proliferation and enhancing production of COL I, proteoglycans, and other ECM components. Fasudil in the TNF family can induce homing of BMSCs [37]. Studies showed that bone morphogenetic protein (BMP)-3, BMP-7, and transforming growth factor (TGF)-β1 can induce BMSCs to differentiate into the NP cell phenotype, and confirmed that appropriate gene transfection can promote the transformation of BMSCs to NP cells [38–40]. In 2003, Sakai et al. [41] first found that autologous BMSCs embedded in atelocollagen gel and transplanted into a rabbit IDD model could delay degeneration of the IVD, and thus studies on BMSCs in IDD were initiated.

Direct and indirect findings from in vitro experiments have shown that BMSCs are beneficial for treatment of IDD. Cao et al. [42] co-cultured BMSCs with NP cells, and found that this combination significantly delayed degeneration of the NP cell matrix by up-regulating TGF-β expression and inhibiting expression of the NF-KB signalling pathway. Fasudil induced actin stress fibre formation by activating the mitogen-activated protein kinase (MAPK) signalling pathway in vitro to promote the migration of rat BMSCs and effectively improve the homing ability of BMSCs [37]. When BMSCs and ADMSCs were separately co-cultured with AF cells for 21 days, the expression of the COL I, COL II, and proteoglycan genes in BMSCs and ADMSCs was increased, and both cell types were able to differentiate into AF cells. Among them, the AF marker COL I was most strongly expressed in the BMSC group, suggesting that BMSCs had stronger ability to differentiate into AF cells and could represent a better cell source for AF repair [43]. When BMSCs were co-cultured with NP cells, the expression of senescence-related genes in NP cells was decreased, i.e. the expression of β-galactosidase was decreased, the synthesis of MMP9 was decreased, and the amount of COL II was increased. At the same time, the expression of zinc metallopeptidase STE24 in NP cells recovered from an inhibition level to the normal level after co-culture, confirming that inhibition of the NF-KB signalling pathway can slow or prevent the aging of NP cells [44, 45]. NCs are key cells for IVD regeneration, and can produce proteoglycans that promote IVD repair. NCs have a stronger ability to tolerate the environment than NP cells, and are thus speculated to be the best seed source for IVD repair. However, NCs disappear when the human body reaches about 10 years of age, which limits their extraction and isolation from the human IVD. Some researchers induced BMSCs from pigs using the NP external matrix and successfully differentiated NCs, providing a new cell source direction for IDD repair by cell transplantation. Indirect evidence confirmed the transformation of BMSCs into NP cells. Regarding co-cultures of BMSCs with NP cells, indirect co-cultures showed that BMSCs produced the NP cell phenotype, while direct co-cultures revealed that BMSCs not only transformed into NP cells, but also formed a similar tunnelling nanotube structure between cell membranes that promoted the phenotypic differentiation of BMSCs. These experiments revealed the relationship between stem cells and NP cells at the level of the molecular mechanism for cell communication [46]. Exosomes (Exos), one of the key messengers for cellular communication, play important roles in material exchange and information transfer between cells. Lu et al. [47] found that exosomes from NP cells (NP-exos) could successfully induce BMSCs to differentiate into NP cells, while exosomes from BMSCs (BMSC-exos) could promote the expression of NP cell-related genes and increase ECM production, thereby achieving the effect of IVD self-repair. BMSC-exos can suppress the NP cell apoptosis induced by TNF-α, and this phenomenon may be related to the transfer of exosome microRNAs targeting activation of the PI3K/AKT pathway [48]. BMSC-exos also inhibit the activation of certain inflammatory mediators and the NLRP3 inflammasome to exert anti-inflammatory effects in NP cells, thereby preventing the occurrence of degenerative changes [49]. Exos have become a new therapeutic approach to reverse IDD due to their low immunogenicity, stable properties, easy acquisition and preservation, and simple transformation. However, due to their disadvantages of low productivity and high heterogeneity, the current applications of exos are limited (Table 2).

**Table 2:** The application of BMSCs in vitro

Year	Team	Source of stem cells	Results
2014	Lehmann et al. [46]	*Human BMSCs* + *Human NP cells*	BMSCs differentiate into NP cells in indirect co-culture group, and form tunneling nanotubes structure which can promote the differentiation of BMSCs in direct co-culture group
2015	Cao et al. [42]	Rabbit BMSCs + rabbit NP cells	NP cells matrix degenerate delay, TGF-β expression↑, inhibit NF-KB signaling pathway
2017	Zhou et al. [43]	Rabbit BMSCs/ADMSCs + AF cells	COL I, COL II and proteoglycan content↑ in both two group, MSCs differentiate into AF cells in both two group, BMSCs perform better than ADMSCs
2017	Lu et al. [47]	Human NP-exos + human BMSCs	BMSCs differentiate into NP cells, ECM↑
2018	Zhan et al. [37]	Rat BMSCs + Fasudil	Activate MAPK signaling pathway, promote BMSCs migration, improve the homing ability of BMSCs
2018	Cheng et al. [48]	Human BMSCs-exos + NP cells	Activate PI3K/AKT signaling pathway to inhibit NP cells apoptosis induced by TNF-α
2019	Li et al. [44]	Rat BMSCs + rat NP cells	COL II content↑, MMP9 synthesis↓, β-galactosidase expression↓, inhibiting NF-KB signaling pathway can slow the aging of NP cells
2019	Li et al. [45]	Pig BMSCs + pig NP cells ECM	BMSCs differentiate into NP cells
2019	Xia et al. [49]	Human BMSCs-exos + NP cells	Inhibit the activation of inflammatory mediators and NLRP3 inflammasome

*ADMSCs* adipose mesenchymal stem cells, *AF* annulus fibrosus, *BMSCs* bone marrow mesenchymal stem cells, *BMSCs-exos* bone marrow mesenchymal stem cells exsomes, *COL I* collagen type I, *COL II* collagen type II, *ECM* extracellular matrix, *MAPK* mitogen activated protein kinase, *MMPs* matrix metalloproteinases, *MSCs* mesenchymal stem cells, *NP* nucleus pulposus, *NP-exos* nucleus pulposus exsomes, *TGF* transforming growth factor, *TNF* tumor necrosis factor

Regarding in vivo experiments, Sakai et al. [50] transplanted autologous BMSCs into a rabbit IDD model, and found that the number of IVD cells increased, the cell survival rate increased, and the proteoglycan content also increased. After injection of human BMSCs into the NP of a bovine IDD model, BMSC apoptosis and migration were regularly detected, and the results showed that the IDD environment did not affect the activity of BMSCs and allowed the cells to migrate to the injured site. At the same time, it was speculated that the stem cells may down-regulate the expression of IL-6, IL-8, and TNF-α through paracrine mechanisms, thus delaying the progression of IDD [35]. An acellular high-density collagen gel loaded with BMSCs was inoculated into the IVD of sheep with AF injury. At 6 weeks after transplantation, the disc height index (DHI), Pfirrmann grade, NP area, and other data were better than those in the control group. Meanwhile, the repair and reconstruction of AF and NP tissues were significantly improved histologically [51]. In terms of efficacy, BMSCs combined with salvianolic acid B had a better effect for IVD repair than BMSCs alone [52]. An IDD model was formed under hydrogen peroxide-induced stress, and then BMSCs and erythropoietin (EPO) were mixed into the IVD of the model. After a period of time, both imaging and histological findings showed that the IVD height and the composition of NP cells were improved. It was also confirmed that EPO could inhibit MSC migration under oxidative stress and stimulate MSC regeneration through local accumulation [53]. Yi et al. [54] obtained transgenic rabbit BMSCs (TBTs) by transfection with a recombinant adenovirus vector carrying human metalloproteinase tissue inhibitor (hTIMP)-1, and implanted them into the rabbit IDD model. Compared with the normal BMSC transplantation group, the degree of IDD in the TBT group was significantly improved, the content of ECM was significantly increased, and the hTIMP-1 mRNA and protein expression levels were higher than those in the normal group. In terms of immune rejection, Shi et al. [55] compared the host immune response to human neonatal and rabbit dermal fibroblasts in a rabbit IDD model, and found that the immune response to human cells implanted into the rabbit IVD was smaller than that to rabbit cells, further indicating that the IVD can tolerate transplantation of allogeneic or xenogeneic cells. Experiments in vivo also indirectly reflected the fact that cell transplantation in combination with loaded genes or drugs may have better therapeutic effects (Table 3).

**Table 3:** The application of BMSCs in vivo

Year	Team	Source of stem cells	Model	Observation time	Results
2006	Sakai et al. [50]	Rabbit BMSCs	Rabbit IDD model	24 weeks	IVD cells↑, cell survival rate↑, proteoglycan content↑
2014	Yi et al. [54]	Transgenic rabbit BMSCs	Rabbit IDD model	12 weeks	IVD degeneration↓, ECM↑, hTIMP-1 mRNA and protein expression levels↑
2018	Teixeira et al. [35]	Human BMSCs	Bovine IDD model	16 days	BMSCs migrate directionally, the expression of IL-6,IL-8,TNF-α ↓ by paracrine stimulation
2019	Hussain et al. [51]	Allogeneic sheep BMSCs + acellular high-density collagen gel	Sheep IDD model	6 weeks	DHI, Pfirrmann grade, NP area all perform well, AF and NP tissue improve in histology
2019	Shi et al. [55]	Neonatal dermal fibroblasts + rabbit dermal fibroblasts	Rabbit IDD model	8 weeks	IVD can tolerate transplantation of allogeneic or xenogeneic cells
2020	Yan et al. [52]	Rabbit BMSCs + salvianolic acid B	Rabbit IDD model	8 weeks	Salvianolic acid B can improve the repair effect of BMSCs
2020	Lykov et al. [53]	Rat BMSCs + EPO	Rat IDD model	21 days	IVD height↑, NP content change, EPO can inhibit the migration of MSCs to prevent IVD repair

*AF* annulus fibrosus, *BMSCs* bone marrow mesenchymal stem cells, *DHI* disc height index, *ECM* extracellular matrix, *EPO* erythropoietin, *hTIMP* metalloproteinase tissue inhibitor, *IDD* intervertebral disc degeneration, *IL* interleukin, *IVD* intervertebral disc, *MSCs* mesenchymal stem cells, *NP* nucleus pulposus, *TNF* tumor necrosis factor

In a clinical study, Orozco et al. [56] injected autologous BMSCs into the NP of ten patients with chronic low back pain caused by lumbar IDD. After 12 months, the patients' low back pain was significantly relieved and the IVD water content was significantly increased on imaging, but the height of the IVD had not changed significantly. Mochida et al. [57] co-cultured NP cells with autologous BMSCs for 1 week, and then implanted the activated NP cells into the IVD adjacent to an interbody fusion segment. Within 3 years of follow-up, they found that the JOA scores had increased, no symptoms of low back pain recurred in all 9 patients, and no adverse reactions after transplantation were observed on imaging. Elabd et al. [58] injected autologous BMSCs cultured under hypoxia into the IVD of five patients with chronic low back pain, and found that the volume of protrusions decreased in four patients during short-term follow-up. In the long-term follow-up, the IVD height in all five patients was maintained or slightly decreased, and the overall condition was improved. Centeno et al. [59] injected autologous BMSCs into the IVD of 33 patients with low back pain and IVD prolapse, and found that this method had significant benefits in alleviating pain, enhancing spinal function, and reducing the IVD prolapse size. Pettine et al. [60] injected autologous BMSCs into the IVD of 26 patients with indications for spinal fusion or IVD replacement. They observed significant improvement in the degree of IDD after 1 year, and only six patients required surgical treatment to improve symptoms within 3 years. Henriksson et al. [61] injected autologous BMSCs into the IVD of four IDD patients. After 8 months, BMSCs were observed to survive in different parts of the IVD and differentiate into chondrocytes, and the original cells were continuously stimulated to produce ECM. Noriega et al. [62] used allogeneic BMSCs to treat 12 patients with chronic low back pain and lumbar IDD who had failed conservative treatment. After 12 months, the VAS, ODI, and Pfirrmann levels in all patients were significantly improved compared with the control group (Table 8).

At present, both autologous BMSCs and allogeneic BMSCs have shown excellent therapeutic effects. Nevertheless, although autologous BMSCs have higher homogeneity, lower treatment cost, wider application, and better therapeutic effect, autologous BMSCs remain the preferred cells in stem cell therapy. However, the method for obtaining BMSCs is invasive, the sample size for clinical treatment is relatively limited, and the long-term efficacy is unknown, warranting further research.

### ADMSCs

ADMSCs, another kind of stem cells with multiple differentiation potential, are more widely derived and can be isolated from adipose tissue. Some studies showed that ADMSCs have stronger differentiation ability than BMSCs and are more suitable for treatment of DDDs [63, 64]. However, there is also evidence that ADMSCs have lower endochondral osteogenic ability than BMSCs [65].

When ADMSCs were cultured with TGF-β3 in vitro, increased amounts of IVD cells and ECM components were observed [66]. At the same time, under induction by TGF-β1, growth differentiation factor (GDF)-5, or GDF-6, ADMSCs had stronger ability to differentiate into NP cells than BMSCs, and the expression levels of ECM components such as sulphated glycosaminoglycans and COL II were significantly higher in the medium [67]. When ADMSCs and NP cells were co-cultured in COL II hydrogel, the number of COL II-secreting NP cells was increased and the expression of the ACAN gene was up-regulated [68]. When rat BMSCs and ADMSCs were separately cultured in a three-dimensional culture system and compared with BMSCs in three-dimensional culture, ADMSCs in three-dimensional culture exhibited higher mRNA and protein expression of NP cell marker genes such as HIF1-α, glucose transporter-1, and chondrocyte-specific genes such as Sox-9, ACAN, and COL II. These findings indicated that ADMSCs had better ability to differentiate into NP cells in three-dimensional culture [69]. In a study on co-culture of ADMSCs and degenerative NP cells, Han et al. [70] found that degenerative NP cells exhibited differential expression of lncRNAs and mRNAs, and subsequently explored the relationship between ADMSCs and NP cells in this co-culture system at the gene expression level. Sun et al. [71] evaluated the effect of ADMSCs on NP cells in a non-physiological mechanical stimulation environment, and found that ADMSCs prevented NP apoptosis by inhibiting caspase-9 and caspase-3, and promoted the TIMP expression by increasing the amount of ECM, thereby inhibiting the effect of MMPs. In addition, the expression of proteoglycans and proinflammatory factors in this system had a positive influence on NP stability. Han et al. [72] co-cultured ADMSCs with degenerative NP cells, and explored the involved signalling pathways and regulatory networks through the lncRNA and mRNA expression profiles in the system. The results showed that ADMSCs in the co-culture system differentially expressed 360 lncRNAs and 1757 mRNAs, had 589 up-regulated genes and 661 down-regulated genes, and showed changes in 299 signalling pathways (Table 4).

**Table 4:** The application of ADMSCs in vitro

Year	Team	Source of stem cells	Results
2008	Lu et al. [68]	Human ADMSCs + NP cells	COL II content↑, ACAN gene expression ↑
2013	Jin et al. [66]	Rat ADMSCs + TGF-β3	IVD cells↑, ECM↑
2014	Clarke et al. [67]	Human ADMSCs + TGF-β1, GDF-5, GDF-6	COL II, glycosaminoglycan content↑, ECM↑,ability of ADMSCs differentiating into NP cells↑
2015	Sun et al. [71]	Human ADMSCs	ECM↑, TIMP expression↑, inhibit caspase-3, caspase-9
2018	Han et al. [70]	Human ADMSCs + degenerative NP cells	lnc RNA and mRNA express differentially
2021	Dai et al. [69]	Rat BMSCs/ADMSCs	ADMSCs perform better in NP cells markers genes and chondrocyte-specific genes expression than BMSCs
2021	Han et al. [72]	Human ADMSCs + degenerative NP cells	Express 360 lncRNA and 1757 mRNA differentially, 589 genes expression↑, 661 expression↓, 299 signaling pathway change

*ACAN* aggrecan, *ADMSCs* adipose mesenchymal stem cells, *BMSCs* bone marrow mesenchymal stem cells, *COL II* collagen type II, *ECM* extracellular matrix, *GDF* growth differentiation factor, *IVD* intervertebral disc, *NP* nucleus pulposus, *TGF* transforming growth factor, *TIMP* metalloproteinase tissue inhibitor

When ADMSCs were injected into a mouse IDD model in vivo, significant positive imaging findings revealed that signals in the IVD were significantly enhanced, with new expression of biglycan and significantly enhanced level of ACAN at 12 weeks after ADMSC implantation [64]. In a study on canine IDD repair, an IDD model was treated with hyaluronic acid (HA) loaded with autologous ADMSCs, and the production of proteoglycans and COL II in the experimental group was significantly increased, indicating that ADMSCs had a stronger stimulating effect on IVD regeneration compared with the control group without ADMSCs [73]. In other experiments, ADMSCs were loaded into acellular NP scaffolds that simulated natural NP components, and the scaffolds were implanted into a rabbit IDD model. The results showed that the combined system had similar mechanical properties and biocompatibility to fresh NP and that the ADMSCs in the scaffolds differentiated into NP cells, while ECM synthesis was also increased [74]. Ishiguro et al. [75] prepared a stent-free tissue engineering structure (TEC) using ADMSCs and applied it to a rat IVD model after NP resection. At 6 weeks after implantation, the TEC showed similar biomechanical characteristics to a sham operation group, while at 6 months, the ADMSC-TEC had not only maintained the height of the IVD, but also retained the AF and CEPs. A major advantage of the TEC-IVD is that it does not degenerate with aging and can thus provide an optimal direction for future treatment of IVD repair. Smoothened Agonist (SAG) can effectively activate the sonic hedgehog (Shh) signalling pathway to promote the differentiation of ADMSCs into NP cells. Hua et al. [76] pre-treated ADMSCs with SAG, as a specific signal agonist of the Shh signalling pathway, and found that the combination of SAG and TGF-β3 significantly increased the gene and protein expression of NP-specific markers at the molecular level as well as the macromolecular level, and significantly changed the IVD height, water content, ECM content, and structure (Table 5).

**Table 5:** The application of ADMSCs in vivo

Year	Team	Source of stem cells	Model	Observation time	Results
2009	Ganey et al. [73]	Canine ADMSCs + HA	Canine IDD model	12 months	COL II, proteoglycan content ↑, cell density↑
2014	Marfia et al. [64]	Human ADMSCs	Rat IDD model	12 weeks	Biglycan content↑, ACAN gene expression↑, imaging signals in IVD↑
2018	Zhou et al. [74]	Human ADMSCs + pig acellular NP scaffold	Rabbit IDD model	16 weeks	ECM↑, the mechanical properties and biocompatibility of the combination system are brilliant, ADMSCs differentiate into NP cells
2019	Ishiguro et al. [75]	Transgenic rat ADMSCs	Rat IDD model without NP	6 months	Biomechanical properties are maintained in 6 weeks, IVD height maintain, AF and CEPs retain in 6 months
2019	Hua et al. [76]	Pretreated human ADMSCs	Rat tail IDD model	16 weeks	NP-specific markers genes expression and ECM↑, IVD height, water content and ECM content all change

*ADMSCs* adipose mesenchymal stem cells, *ACAN* aggrecan, *AF* annulus fibrosus, *CEPs* cartilaginous endplates, *COL II* collagen type II, *ECM* extracellular matrix, *HA* hyaluronic acid, *IDD* intervertebral disc degeneration, *IVD* intervertebral disc, *NP* nucleus pulposus

In a clinical study, Kumar et al. [77] implanted a mixture of HA and ADMSCs into the IVD of ten IDD patients. At the 1-year follow-up, no adverse reactions were found and more than one-half of the patients showed significant improvement in the VAS and ODI, with only three patients showing an increased IVD water content on imaging (Table 8).

Like BMSCs, ADMSCs also have the ability to differentiate into NP cells, produce different growth factors, and play a role in IVD repair at different levels. ADMSCs have a wide range of sources and are easy to obtain, providing unique advantages over other stem cells and having bright prospects for application. However, the number of clinical trials is not currently sufficient to support the long-term effectiveness and safety of the technology.

### UCMSCs

UCMSCs mainly exist in Wharton's Jelly of the umbilical cord, and are also known as Wharton’s Jelly mesenchymal stem cells (WJMSCs). Although these stem cells are not limited by ethical barriers, they cannot be obtained autologously for IDD patients. Compared with most other MSCs, WJMSCs are a relatively young type of MSCs with strong proliferation ability, extensive differentiation ability, low immunogenicity, and no tumorigenicity [78–80].

The gene expression pattern in WJMSCs in vitro is similar to that in ADMSCs, and can be regulated by inflammatory stimulation [81]. Beeravolu et al. [82] induced differentiation of WJMSCs into chondrogenic progenitor cells (CPCs) in vitro, and found that the cell structure, ECM, and glycosaminoglycan content of CPCs were significantly improved compared with original WJMSCs. Other experiments showed that WJMSCs significantly increased the expression of proteoglycans and COL II in the NP, thus promoting ECM formation. WJMSCs were able to regulate mitosis and apoptosis by up-regulating expression of Bcl-2 and down-regulating expression of Bax, thereby protecting NP cells against high glucose damage [83]. Meanwhile, WJMSCs mitigated ECM degradation in a high glucose environment through the P38 MAPK signalling pathway, producing conditions conducive to IVD repair. Compared with IDD patients showing mild degeneration, caspase-3, Bax, Wnt3a, Wnt5a, Wnt10a, GSK-3β, cyclin-D1, and β-catenin expression was significantly increased with IDD progression, while Bcl-2 expression was significantly decreased in patients with severe IDD. Zhao et al. [84] isolated NP cells from patients with mild or severe IDD, induced apoptosis by compression, and conducted co-cultures with WJMSCs. They found that the apoptotic ratio of NP cells increased, but was significantly reversed on co-culture with WJMSCs. Meanwhile, WJMSCs also blocked the Wnt/β-catenin pathway through DKK-1 to inhibit the expression of Wnt-related genes, thus guaranteeing the anti-apoptotic effect of WJMSCs (Table 6).

**Table 6:** The application of WJMSCs, IVDSCs and PSCs in vitro

Year	Team	Source of stem cells	Results
2016	Wang et al. [81]	Human WJMSCs	WJMSCs genes expression pattern is similar to ADMSCs, inflammatory stimulation can regulate genes expression
2018	Beeravolu et al. [82]	Induce human WJMSCs differentiating into CPCs	Cell structure change, glycosaminoglycan content and ECM↑
2019	Qi et al. [83]	Human WJMSCs	Activate MAPK signaling pathway, promote B-cell lymphoma-2 expression and inhibit Bax expression, ECM↑and ECM degradation ↓, COL II and proteoglycan content↑, regulate mitosis and apoptosis
2020	Zhao et al. [84]	Human WJMSCs + human NP cells	Reverse the apoptotic ratio of NP cells, block Wnt/β-catenin pathway to inhibit Wnt-related genes expression
2013	Barreto et al. [85]	Rabbit NPSCs	NPSCs migrate along the fibers and cells in IVD
2016	Wang et al. [86]	BMSCs/NPSCs/AFSCs/CESCs	CESCs are stronger in osteogenesis and chondrogenesis than the other three
2017	Huang et al. [87]	NPSCs	Hypoxia can promote NPSCs proliferation related to HIF-1α signaling pathway, Simvastatin can promote NPSCs transfer into NP cells
2018	He et al. [88]	Human CESCs	Promote CEPs repair, promote NP cells proliferation
2015	Liu et al. [89]	Human IPSCs + pig NP cell ECM	NP cells secret regulatory factors, mediate the differentiation of IPSCs into NP cells
2020	Hu et al. [90]	GDF-5 transfected human IPSCs + rat NP cells	Chondrocyte markers genes expression↑

*ADMSCs* adipose mesenchymal stem cells, *AFSCs* annulus fibrosus stem cells, *BMSCs* bone marrow mesenchymal stem cells, *CEPs* cartilaginous endplates, *CESCs* cartilage endplate stem cells, *COL II* collagen type II, *CPCs* chondrogenic progenitor cells, *ECM* extracellular matrix, *GDF* growth differentiation factor, *HIF* hypoxia inducible factor, *IPSCs* induced pluripotent stem cells, *IVD* intervertebral disc, *MAPK* mitogen activated protein kinase, *NP* nucleus pulposus, *NPSCs* nucleus pulposus stem cells, *WJMSCs* Wharton's Jelly mesenchymal stem cells

In an in vivo study, McKee et al. [91] loaded WJMSCs and rabbit degenerative NP cells into a specific scaffold and conducted experiments in a rabbit IDD model. They found that this combination not only promoted the transformation of WJMSCs into NP cells, but also prevented cell leakage during and after implantation, avoided complications, and achieved the effect of IVD repair by transplantation of stem cells. After WJMSCs were induced to differentiate into CPCs and implanted into a rabbit IDD model, NP-specific markers were more significantly expressed in the CPC group compared with the WJMSC group [82] (Table 7).

**Table 7:** The application of WJMSCs, IVDSCs and PSCs in vivo

Year	Team	Source of stem cells	Model	Observation time	Results
2018	Beeravolu et al. [82]	WJMSCs differentiate into CPCs	Rabbit IDD model	8 weeks	NP-specific markers genes express more significantly in CPCs group
2020	McKee et al. [91]	Human WJMSCs + degenerative rabbit NP cells + scaffold	Rabbit IDD model	8 weeks	Promote WJMSCs differentiating into NP cells, avoid cell leakage and complications
2016	Chen et al. [92]	Human NPSCs/NP cells	Rabbit IDD model	8 weeks	NPSCs have better IVD repair effect than NP cells
2021	Marimuthu et al. [93]	Rat NPSCs	Allogeneic rat IDD model	21 days	NPSCs regenerative ability is stronger, MSCs anabolic activity↑and catabolic activity ↓, allotransplantation cause pro-inflammatory effect stronger
2009	Sheikh et al. [94]	Rat ESCs	Rabbit IDD model	8 weeks	Generate NCs
2020	Hu et al. [90]	Transfected human IPSCs + GDF-5 hydrogel	Rat IDD model	3 months	IDD symptoms improve both in imaging and histology
2021	Sun et al. [95]	Transfect human IPSCs to IMSCs	Rat IDD model	8 weeks	NP cells senescence delay, and restore the age-related function, activate SIRT6 pathway, downregulate PDE4D level

*CPCs* chondrogenic progenitor cells, *ESCs* embryonic stem cells, *GDF* growth differentiation factor, *IDD* intervertebral disc degeneration, *IMSCs* induced mesenchymal stem cells, *IVD* intervertebral disc, *IPSCs* induced pluripotent stem cells, MSCs mesenchymal stem cells, *NCs* notochord cells, *NP* nucleus pulposus, *NPSCs* nucleus pulposus stem cells, *WJMSCs* Wharton's Jelly mesenchymal stem cells

In a clinical study, two patients with chronic discogenic low back pain were treated with IVD injection of WJMSCs. During follow-up for 2 years, the VAS and ODI in both patients were significantly improved, and their pain symptoms were significantly alleviated [96] (Table 8).

**Table 8:** The application of stem cells in clinic

Year	Team	Types of cells	Number of cases	Observation time	Results
2011	Orozco et al. [56]	Autologous BMSCs	10	12 months	IVD water content↑, IVD height do not change
2015	Mochida et al. [57]	Autologous BMSCs + NP cells	9	3 years	JOA↑ in 9 patients, no symptoms of low back pain, no adverse reactions
2016	Elabd et al. [58]	Autologous BMSCs under hypoxia	5	Long-term	Protrusions volume↓in 4 patients, IVD height keep or slightly↓
2017	Centeno et al. [59]	Autologous BMSCs	33	6 years	Pain↓, spinal function↑, disc prolapse↓
2017	Pettine et al. [60]	Autologous BMSCs	26	3 years	IVD function↑ in all patients in 1 year, only 6 patients need surgical treatment in 3 years
2017	Noriega et al. [62]	Allogeneic BMSCs	12	12 months	VAS, ODI and Pfirrmann levels all perform well
2019	Henriksson et al. [61]	Autologous BMSCs	4	8 months	BMSCs differentiate into chondrocytes, ECM↑
2017	Kumar et al. [77]	Human ADMSCs + HA	10	12 months	VAS and ODI perform better over 5 patients, IVD water content↑ in 3 patients, no adverse reactions
2014	Pang et al. [96]	Human WJMSCs	2	2 years	VAS, ODI perform well

*ADMSCs* adipose mesenchymal stem cells, *BMSCs* bone marrow mesenchymal stem cells, *DHI* disc height index, *ECM* extracellular matrix, *HA* hyaluronic acid, *IVD* intervertebral disc, *JOA* Japanese Orthopaedic Association scores, *NP* nucleus pulposus, *ODI* Oswestry Disability Index, *VAS* visual analogue scale, *WJMSCs* Wharton's Jelly mesenchymal stem cells

WJMSCs are rarely used in the treatment of IDD. Nevertheless, experiments have confirmed that WJMSCs can be induced to differentiate into CPCs and then transplanted in vivo, leading to not only an enhanced repair effect relative to WJMSC transplantation, but also avoidance of adverse consequences resulting from pluridirectional differentiation of stem cells as much as possible. At present, however, the experimental cost of WJMSCs is high, and WJMSCs do not appear to have outstanding advantages over other MSCs. Although there have been few relevant reports in recent years, it is undeniable that practical application of WJMSCs in the treatment of IDD has feasibility and potential.

### IVDSCs

Previous studies demonstrated the existence of a certain number of MSCs, namely NPSCs, AFSCs, and CESCs, in the NP, AF, or CEPs of the IVD, which are collectively called IVDSCs. These cells share the characteristics of stem cell growth and differentiation, and thus have potential for IVD repair applications.

In an in vitro study, Wang et al. [86] compared the proliferation ability of BMSCs, NPSCs, AFSCs, and CESCs, and found that CESCs had the strongest ability for osteogenesis and chondrogenesis among these cell types. Other studies showed that CESCs can promote not only repair of CEPs, but also proliferation of NP cells through paracrine mechanisms [88]. NPSCs in NP tissues can migrate along the fibres and cells in the IVD [85]. Meanwhile, hypoxia can promote proliferation of NPSCs, which may be related to the HIF-1α signal transduction pathway mediated by SIRT1 and SIRT6. Simvastatin can promote the expression of hypoxia-inducible factors and thus induce the differentiation of NPSCs into NP cells, achieving the effect of IVD repair [87] (Table 6).

Chen et al. [92] compared the in vivo therapeutic effect of NPSCs and NP cells transplanted into a rabbit IDD model, and found that the former had a better effect for IVD repair on imaging evaluation. Marimuthu et al. [93] isolated and amplified NP tissues from the IVD of normal mice, cultured NPSCs, and carried out allotransplantation. The results showed that NPSCs had good regenerative ability, and that this NP cell-based treatment led to enhanced anabolic activity of MSCs, while their catabolic activity was weakened. It was further found that NPSCs from allogeneic sources had a stronger stimulating effect on secretion of pro-inflammatory factors than NPSCs from homogeneic sources (Table 7).

At present, the application of IVDSCs faces great challenges. Although CESCs have the best repair effect, their efficiency for separation from CEPs is extremely low. In addition, it is difficult to specifically induce CESCs to differentiate into hyaline cartilage tissue. The microenvironment of the IVD is characterized by high pressure, high osmosis, low oxygen, low pH, and insufficient nutrient supply, and the occurrence of IDD makes the microenvironment more severe. Furthermore, IVDSCs will age and undergo denaturation. These changing conditions pose a challenge to the viability of implanted stem cells.

### PSCs

PSCs, with high capacities for self-renewal, proliferation, and differentiation, include IPSCs and ESCs. IPSCs are a special kind of cells generated by inducing the expression of ectopic recombinant transcription factors. For example, IPSCs derived from NP tissues can be induced to differentiate into NP-like chondrocytes that possess strong self-renewal, proliferation, and differentiation abilities. However, IPSCs are potentially tumorigenic. ESCs are pluripotent stem cells derived from discarded frozen early embryos. Currently, there are limited cell lines available for studies due to ethical issues. ESCs can differentiate into all derivatives of the three primary germ layers [11, 97].

In an in vitro study, Liu et al. [89] found that NP cells can secrete some regulatory factors that induce stem cells, and that these regulatory factors can promote differentiation of IPSCs into NP-like cells in the ECM, thus achieving the purpose of IVD repair. When GDF-5-transfected human IPSCs (GDF5-hIPSCs) were co-cultured with rat NP cells in vitro, the mRNA expression of three chondrocyte markers, SOX9, COL II, and proteoglycans, was significantly increased [90] (Table 6).

In an in vivo study, a GDF5-hIPSC hydrogel prepared by Hu et al. [90] was implanted into a rat IDD model. It was found that this complex gel effectively improved the symptoms of IDD by imaging and histological findings at 1, 2, and 3 months. When ESC-derived chondrocyte progenitors from mice were transplanted into the degenerative IVD of rabbits, NCs were successfully generated and proteoglycans were then produced by these NCs to promote IVD repair [94]. Use of MSC-derived small extracellular vesicles (MSC-SEVs) is a novel strategy for the treatment of IDD. However, due to the disadvantages of MSCs, such as limited proliferation ability and invasive acquisition, the application and development of MSC-SEVs are limited. IMSCs derived from IPSCs can provide an abundant source of MSC-SEVs. After injection of IMSC-SEVs into an IDD model, the senescence of NP cells was significantly delayed and the IDD status was obviously improved. IMSC-SEVs rejuvenated the senescent NP cells and restored the age-related function by activating the SIRT6 pathway in vitro. Furthermore, microRNA sequence analysis showed that IMSC-SEVs were highly enriched in miR-105-5p, which had a pivotal role in the IMSC-SEV-mediated therapeutic effect by downregulating the level of cAMP-specific hydrolase PDE4D and leading to SIRT6 activation [95] (Table 7).

**Fig. 3:**
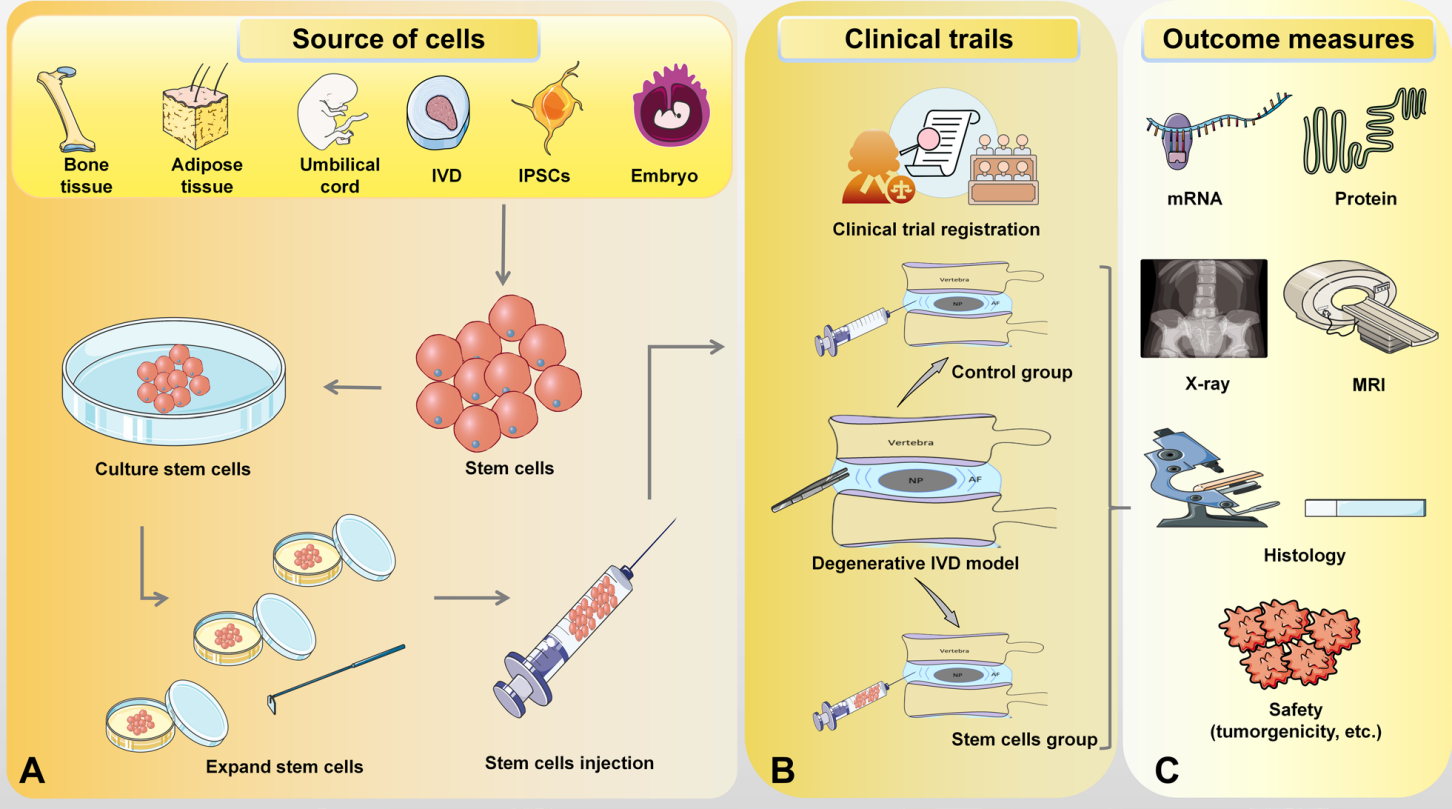
Diagram of stem cell therapy for IVD degeneration. (**A**) Stem cell acquisition in vitro. (**B**) Stem cells and control group inject into degenerative IVD in vivo. (**C**) Tools to detect the role of stem cells in the degenerative disc. *IPSCs* induced pluripotent stem cells, *IVD* intervertebral disc

Although PSCs can transform into the NP cell phenotype and promote repair in IDD, IPSCs have the characteristics of tumour cell proliferation and differentiation, and the directional differentiation of ESCs in vitro is unstable and inherently carcinogenic. These shortcomings as well as legal and ethical problems limit the further application of these two cell types (Fig. 3).

### Defects in the application of stem cells

Although many reports have demonstrated novel advantages of different stem cells in the treatment of IDD, other reports have described deficiencies in the application of stem cells. In addition to the disadvantages already mentioned, Coric et al. [98] found that cell injection therapy exceeding the normal range of cell dose was ineffective in delaying IDD or led to worse outcomes. Local injection of excessive numbers of cells may cause cell accumulation and death, thus triggering an inflammatory response. When injecting stem cells, the amount of injection is of great concern. Haufe et al. [99] used hematopoietic stem cells (HSCs) to conduct experiments on IDD in animal models and patients. While the animal experiments showed that the IVD could be regenerated by injection of HSCs, none of the ten patients had significantly improved symptoms of discogenic low back pain at the 6-month and 1-year follow-up visits. Therefore, the selection of stem cells plays a decisive role in the treatment of different subjects. Meisel et al. [8] analysed small case studies and found that stem cell therapy for patients with IDD may be useful in alleviating pain or improving IVD function, but the overall data on efficacy and safety did not reveal any major findings, and it was not clear whether the changes in symptoms were clinically important. Although the clinical efficacy was significant, there was a large risk of bias. In addition, at the stage of cell injection therapy, MSCs may undergo unnecessary cell migration or cell leakage after IVD injection, resulting in ineffective treatment and osteophyte formation [100]. Therefore, cell carriers and AF sealing technologies are particularly important. Certainly, an improper puncture operation can lead to disk infection or diskitis [101].

## Stem cell vectors in development

**Fig. 4:**
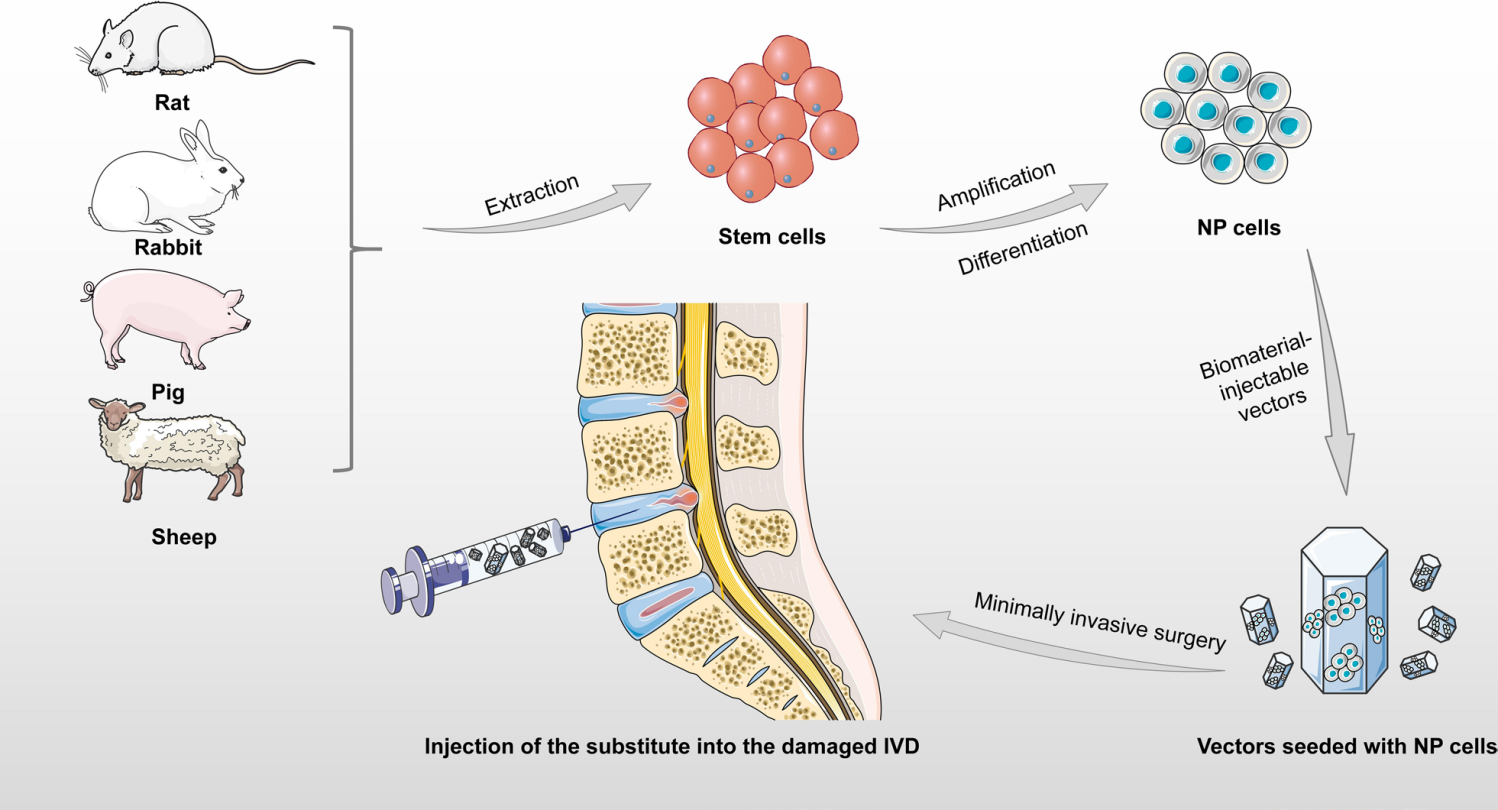
Diagram of the application of stem cell vectors. IVD intervertebral disc; NP nucleus pulposus

In the application of stem cell transplantation, not only do the growth and differentiation degree of transplanted cells affect the therapeutic effect, but also the leakage of stem cells at the implantation site hinders the development of stem cells. Therefore, it is particularly important to control adverse cell migration and differentiation during IDD stem cell injection therapy. Many studies have shown that certain biomaterials can provide three-dimensional structures suitable for stem cell growth to control undesirable migration, and can simultaneously be skilfully combined with biological factors or drugs that promote the differentiation of stem cells to NP cells, thereby promoting ECM secretion, inducing MSC differentiation to the NP cell phenotype, and accelerating IDD repair [91, 102, 103] (Fig. 4).

Vadala et al. [100] injected rabbit autologous BMSCs into an IDD model. They found no obvious IVD regeneration and no labelled MSCs in the IVD, but observed that the injected stem cells migrated to the outside of the IVD and formed undesirable osteophytes. To solve such adverse problems, researchers have considered different approaches, such as the co-culture stage, drug-loaded injection, and cell differentiation stage in vivo, to combine stem cells with different vectors that avoid the occurrence of adverse reactions, and ensure that stem cells can continue to play a stable, efficient, and accurate role.

BMSCs embedded in alginate microcapsules were implanted into the NP of the bovine IVD in vitro after low temperature and low oxygen disposal, and this co-culture was found to lead to increased production of ECM components [104]. Co-culture of mouse BMSCs with glucose-gelatine hydrogel scaffold-loaded TGF-β3 effectively induced the differentiation of BMSCs into NP cells and promoted the expression of ECM-related genes [105]. When microencapsulated rabbit chondrocytes were co-cultured with BMSCs, the activity of BMSCs and the expression of COL II and proteoglycans were detected. The microcapsule co-culture system not only had no effect on the function of BMSCs, but also increased the production of COL II and proteoglycans. At the same time, an in vivo experiment showed that the repair effect in the co-culture group was significantly better than that in the monoculture BMSC group [106]. The functional self-assembled peptide nanofibre scaffold prepared by Wu et al. [107] was proven to promote the differentiation, proliferation, and chemotactic migration of BMSCs to NP cells in experiments. Kuang et al. [108] cultured NPSCs into a hydrogel scaffold made of acellular NP matrix and chitosan, and then added TGF-β3. Compared with the simple hydrogel group, NPSCs grew better in the mixed hydrogel group containing growth factors, and the COL I, COL II, and proteoglycan genes were more strongly expressed. An HA-methylcellulose hydrogel system loaded with WJMSCs was implanted into a rat IDD model. Compared with WJMSCs alone, the combined group had a stronger IVD repair effect by improving the viability of NP cells and reducing the degradation of ECM [109]. There is also a hot spot in current research for loading drugs into the body through carriers and producing corresponding effects on stem cells. For example, injection of albumin/heparin nanoparticles loaded with stromal cell-derived factor SDF-1α into an IDD model induced IVD regeneration and enhanced the homing ability of BMSCs [110] (Table 9).

**Table 9:** The application of Stem Cell Vectors

Year	Team	Source of stem cells and vectors	Model	Results
2016	Gan et al. [105]	Glucose-gelatin hydrogel scaffold-loaded TGF-β3 + mouse BMSCs	In vitro	BMSCs differentiate into NP cells, ECM gene expression↑
2016	Wu et al. [107]	Self-assembled peptide nanofiber scaffold + rabbit BMSCs + rabbit chondrocytes	Rabbit IDD model	Promote BMSCs differentiating into NP cells, promote BMSCs proliferation and chemotactic migration
2018	Zhang et al. [106]	Microencapsulated rabbit chondrocytes + rabbit BMSCs	Rabbit IDD model	The combination system do not affect the function of BMSCs, COL II and proteoglycan content↑, the repair effect of co-culture group perform better than the original BMSCs group
2018	Zhang et al. [110]	Albumin/heparin nanoparticles loaded with SDF-1α	Rabbit IDD model	Induce IVD regeneration, enhance the homing ability of BMSCs
2018	Zhou et al. [74]	Human ADMSCs loaded in pig acellular NP scaffolds	Rabbit IDD model	ECM↑, the mechanical properties and biocompatibility of the combination system are brilliant, ADMSCs differentiate into NP cells
2019	Hussain et al. [51]	Allogeneic sheep BMSCs loaded in acellular high-density collagen gel	Sheep IDD model with AF injured	DHI, Pfirrmann grade, NP area all perform well in 6 weeks, AF and NP tissue improve in histology
2019	Naqvi et al. [104]	Pig BMSCs embedded in alginate microcapsules	Bovine IVD model	ECM↑
2019	Ishiguro et al. [75]	Stent-free tissue engineering structure made by ADMSCs	Rat IDD model without NP	Biomechanical properties are maintained, IVD height maintain, AF and CEPs retain
2020	Choi et al. [109]	WJMSCs loaded in hyaluronic acid-methylcellulose hydrogel system	Rat IDD model	Improve the viability of NP cells, reduce ECM degradation
2021	Kuang et al. [108]	Acellular NP matrix and chitosan hydrogel scaffold + TGF-β3 + NPSCs	In vitro	NPSCs grow better in scaffold group, COL I,COL II and proteoglycan content↑

*ADMSCs* adipose mesenchymal stem cells, *AF* annulus fibrosus, *BMSCs* bone marrow mesenchymal stem cells, *CEPs* cartilaginous endplates, *COL I* collagen type I, *COL II* collagen type II, *DHI* disc height index, *ECM* extracellular matrix, *IDD* intervertebral disc degeneration, *IVD* intervertebral disc, *NP* nucleus pulposus, *NPSCs* nucleus pulposus stem cells, *SDF* stromal cell-derived factor, *TGF* transforming growth factor, *WJMSCs* Wharton's Jelly mesenchymal stem cells

## Conclusions

Whether examined in vitro, in animals, or in clinical trials, stem cell therapy for DDDs has been proven to show promise in different aspects. Stem cells from different sources have different degrees of ability to induce differentiation, produce different biological effects, and promote the expression of different biological factors. With the deepening of stem cell research, our understanding of stem cell therapy for IDD has improved. Meanwhile, the indications for stem cell therapy for IDD have gradually expanded, and the sources of stem cells have been enriched, laying a solid foundation for the experimental and clinical application of stem cell therapy for IDD.

Although cell therapy appears to have great potential for IVD regeneration, there remains a lack of relevant evidence regarding safety, long-term complications, effectiveness in different patient populations, and surgical cost-effectiveness. Further development of stem cell technology and in-depth exploration of IDD in the medical community will determine the future development direction of the organic combination of stem cells and IDD research. First, we need to further explore the interactions between stem cell repair mechanisms and target cells, and strive to identify more targets that promote differentiation. Second, we need to find ways to improve the harsh microenvironment in IDD to provide a better living environment for loaded stem cells. Third, we need to establish methods that can induce and differentiate stem cells from different sources more efficiently and stably, thereby improving the safety of stem cell application. Last, but not the least, it is necessary to optimize the performance of stem cell carrier materials to avoid secondary damage during implantation and further enhance the repair ability of stem cells.

## Data Availability

Not applicable.

## Abbreviations

ACAN: Aggrecan
ADMSCs: Adipose mesenchymal stem cells
AF: Annulus fibrosus
AFSCs: Annulus fibrosus stem cells
BMP: Bone morphogenetic protein
BMSCs: Bone marrow mesenchymal stem cells
CEPs: Cartilaginous endplates
CESCs: Cartilage endplate stem cells
COL I: Collagen type I
CPCs: Chondrogenic progenitor cells
DDDs: Degenerative disc diseases
DHI: Disc height index
ECM: Extracellular matrix
EPO: Erythropoietin
ESCs: Embryonic stem cells
Exos: Exsomes
GDF: Growth differentiation factor
GDF-5-hIPSCs: GDF-5-transfected human IPSCs
HA: Hyaluronic acid
hTIMP: Metalloproteinase tissue inhibitor
IDD: Intervertebral disc degeneration
IFN: Interferon
IL: Interleukin
IPSCs: Induced pluripotent stem cells
IVD: Intervertebral disc
IVDSCs: Intervertebral-derived stem cells
MAPK: Mitogen activated protein kinase
MMPs: Matrix metalloproteinases
MSCs: Mesenchymal stem cells
MSCs-SEVs: Derived small extracellular vesicles
NCs: Notochord cells
NP: Nucleus pulposus
NPSCs: Nucleus pulposus stem cells
PGE: Prostaglandin E
PSCs: Pluripotent stem cells
SAG: Smoothened agonist
Shh: Sonic hedgehog
TBT: Transgenic BMSCs
TEC: Tissue engineering structure
TNF: Tumor necrosis factor
UCMSCs: Umbilical cord mesenchymal stem cells
WJMSCs: Wharton's Jelly mesenchymal stem cells
